# The Improved Abscess Syringe

**Published:** 1893-04

**Authors:** 


### The Improved Abscess Syringe.

We have had in use for some time one of Dr. J. A. Dunn's Improved Abscess Syringes. In several respects it is quite an improvement over the one formerly made. The body of the syringe is of glass to which an improved rubber bulb is attached; a vulcanized rubber collar slips closely on to the end of the glass cylinder. Through this rubber collar extends a hollow tube or needle of gold or platinum. This makes a very perfect instrument for applying any liquid preparation in the treatment of abscesses. Acids will not act, of course, upon any of its parts; the glass, the vulcanized rubber, and the needle are all proof against the action of any acid that is used in such treatment.

It is not easy to conceive of an instrument better adapted to the purposes specified than this one of Dr. Dunn's, and it should be in the hands of every dentist.

